# Partition of Omega-like facility into two configurations of 24 and 36 laser beams to improve implosion performance

**DOI:** 10.1038/s41598-023-37174-1

**Published:** 2023-06-20

**Authors:** M. Temporal, A. R. Piriz, B. Canaud, R. Ramis, R. S. Craxton

**Affiliations:** 1 Instituto de Investigaciones Energéticas (INEI), E.T.S.I.I., and CYTEMA, Universidad de Castilla-La Mancha, 13071 Ciudad Real, Spain; 2 CEA, DAM, DIF, 91297 Arpajon, France; 3 Université Paris-Saclay, CEA, LMCE, 91680 Bruyères-Le-Châtel, France; 4 ETSI Aeronáutica y del Espacio, Universidad Politécnica de Madrid, 28040 Madrid, Spain; 5 Laboratory for Laser Energetics, University of Rochester, Rochester, NY 14623-1299 USA

**Keywords:** Nuclear fusion and fission, Laser-produced plasmas

## Abstract

An Omega-like beam configuration is considered where the 60-beam layout can be separated into two independent sub-configurations with 24 and 36 laser beams, each minimizing direct drive illumination non-uniformity. Two different laser focal spot profiles, one associated with each configuration, are proposed to apply the zooming technique in order to increase the laser-target coupling efficiency. This approach is used by 1D hydrodynamics simulations of the implosion of a direct-drive capsule characterized by a relatively large aspect ratio A = 7 and an optimized laser pulse shape delivering a maximum of 30 TW and 30 kJ, with different temporal pulse shapes in each of the two sets of beams. It is shown that zooming allows for an optimistic 1D thermonuclear energy gain greater than one while without zooming the thermonuclear gain remains largely below one. While this is incompatible with the as-built Omega laser, it provides a promising option for a future intermediate-energy direct drive laser system.

## Introduction

One of the main goals of Inertial Confinement Fusion (ICF)^1–4^ is the compression and heating of a relatively small amount of Deuterium–Tritium (DT) thermonuclear fuel contained in a spherical capsule. A successful capsule implosion should generate a hot-spot characterized by a high temperature where the nuclear fusion reaction D + T → α + n + 17.6 MeV will take place. The driver usually consists of several high-power laser beams although others drivers have also been considered^5–8^. Several laser facilities are already operating: the 192 laser beams (1.8 MJ) of the National Ignition facility (NIF, Livermore-USA) organized in 48 quads^9,10^, a subset (64 beams) of the 176 laser beams (44 quads, 1.3 MJ) of the LMJ^11^ (Bordeaux-France), the 48 laser beams (180 kJ) of the SG-III facility^12^ (Shenguang-China), the 60 laser beams (30 kJ) of the Omega facility^13^ (Rochester-USA), the 12 laser beams of Gekko XII^14^ (Osaka-Japan), and the 10 laser beams of the Orion facility^15^ (Berkshire-United Kingdom). Ignition of nuclear fusion reactions is not the only purpose of these installations, which also offer a way to compress matter and access high-energy–density states of interest, e.g. warm dense matter^16^, astrophysics^17^, or particle acceleration^18^.

Concerning the ignition of DT nuclear fusion reactions there are two main approaches: indirect-drive^2^ and direct-drive^19–21^. In the indirect-drive approach the laser beam energy is first converted in a high-Z cavity into a uniform X-ray field that irradiates the spherical capsule. In contrast, in the direct-drive approach the laser beams irradiate the external shell (absorber) of the capsule. Both approaches launch a series of shock waves that induce the implosion process. Indirect drive is less efficient but provides more robust capsule implosions due to the highly uniform irradiation of the target provided by the X-ray field, higher ablation rate and consequent better ablative stabilization of hydrodynamic instabilities. Nevertheless, for both schemes the uniformity of the irradiation is an important issue. Irradiation non-uniformity can induce deformations of the shell and seed the growth of dangerous hydrodynamic instabilities (Richtmyer–Meshkov and Rayleigh–Taylor^22–24^) that can prevent the success of the implosion. Moreover, low mode deformation gives rise to residual kinetic energy^25^ in the assembled fuel, reducing stagnation efficiency. The nuclear fusion reactions are generated in a small portion of the DT fuel, the hot-spot. Once the ignition conditions (ρR > 0.3 g/cm^2^ and T ≈ 10 keV) are generated, where ρR is the density-radius product of the fuel and T is the hot-spot temperature, a thermonuclear burn wave should propagate through the DT mass providing high energy gain. The burn-up fraction is approximately given by *f* = ρR/(ρR + 7 g/cm^2^), where ρR is the maximum areal mass, so an areal mass of ρR = 3 g/cm^2^ is needed for the combustion of about 1/3 of the DT fuel. The energy gain is given by G = 3.4 10^11^ m_DT_ *f* (η / E_a_), with the fuel mass m_DT_ in [g], the absorbed laser energy E_a_ in [J] and the laser absorption η = E_a_ / E_L_, the ratio between the incident (E_L_) and the absorbed laser energy. Thus, the gain increases with the laser absorption fraction, which—for the direct-drive approach—depends strongly on the dimension of the laser focal spot. One possibility to increase the laser absorption is to use zooming^26,27^. The idea is to adjust the laser focal spot at different stages of the capsule implosion in order to increase the total laser-plasma coupling. The laser pulse is usually composed of a low-power pre-pulse followed by a high-power main drive. The laser focal spot optimized for the first few ns of the implosion is larger than the one required for the main pulse because the radius of the critical surface and the radii at which laser light is absorbed are reduced as the implosion proceeds, so that spots tuned to the initial capsule size will overfill the capsule at late time.

This paper considers the possibility of adapting the zooming technique to an Omega-like facility^28^. First, it is shown that a 60-laser-beam configuration identical to that used for the Omega facility can be separated into two direct-drive configurations of 24 and 36 laser beams and that both configurations would provide relatively small illumination non-uniformity. This suggests separating the full laser pulse into two parts: a first pulse dedicated to the fuel assembly and a second more powerful pulse to assist the fuel ignition, much as in shock ignition^29^. The zooming technique associates two different laser focal spots to the two pulses in order to increase the laser-plasma coupling. While this concept cannot be applied to the as-built Omega laser because the two sets of beams cannot be given independent pulse shapes, a parametric study has been performed for a system built without this constraint but still broadly remaining within the limits of the laser energy and power of the Omega facility. Designs have been found producing an energy gain larger than unity. These designs can be scaled up to future laser systems delivering greater energy but using the same Omega geometry.

### Partition of an Omega-like facility into two configurations with 24 and 36 laser beams

**Figure 1:**
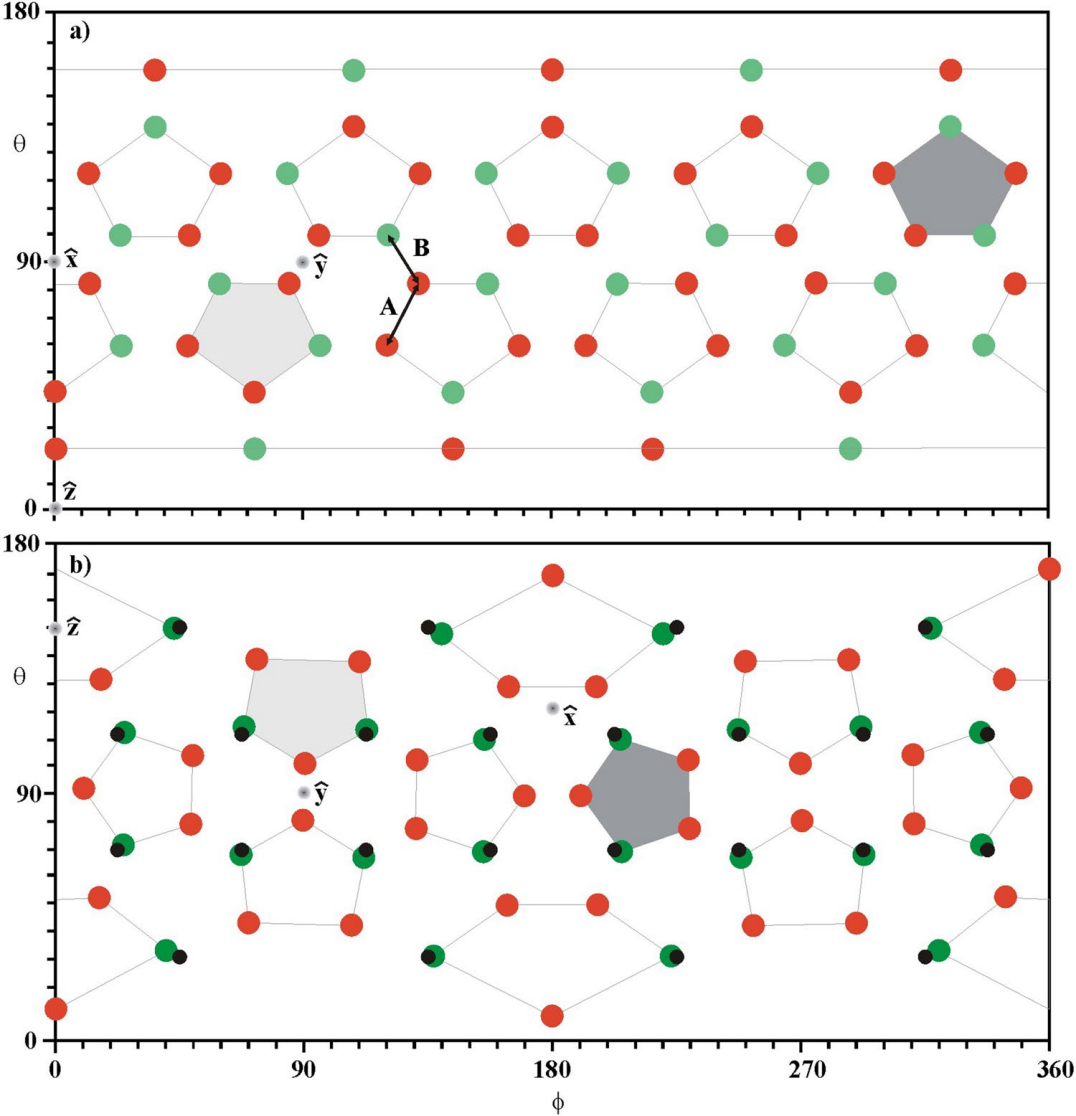
(**a**) 60-beam configuration of the Omega facility, the green circles indicating the 24-beam set and the red circles the 36-beam set. (**b**) The same beams after a suitable rotation around the y axis. The locations of the 24 beams in the configuration of Skupsky and Lee are shown by the smaller black circles.

The Omega facility (formerly known as the Omega Upgrade)^13,28^ uses 60 laser beams distributed in a highly symmetrical configuration based on the dodecahedron platonic solid (12 pentagonal faces and 20 vertices). This is used to construct a truncated icosahedron with 12 pentagonal and 20 hexagonal faces. The laser beams of the Omega facility are located at the 60 vertices of the 12 pentagons of the truncated icosahedron characterized by a stretching factor of A / B = 1.2, the ratio between the edges A and B shown in Fig. 1a. Since the polar axis corresponds to the centre of a pentagon, the 30 beams of each hemisphere are located on four rings^13,28,39^, namely: 5 beams at a polar angle of 21.415^°^, 5 beams at 42.020^°^, 10 beams at 58.852° and 10 beams at 81.249^°^. This is the usual representation of the 60 beams of the Omega facility, with the beam locations in terms of latitude θ ∈ [0–180^°^] and longitude ϕ ∈ [0–360^°^] given in Fig. 1a, where it is possible to recognize the 12 pentagons that are characteristics of the 60-beam configuration (fullerene—C60 molecule).

In this representation the polar z-axis has coordinates (θ = 0, ϕ = 0), the x-axis (θ = π/2, ϕ = 0) and the y-axis (θ = π/2, ϕ = π/2). It can be seen that a rotation of π–θ_1_ around the y-axis almost re-allocates 24 beams into two rings per hemisphere, namely 4 beams are close to the ring at the polar angle θ_1_ = 30.361° and the other 8 beams are closer to the ring at θ_2_ = 69.059^°^. Figure 1b shows the new locations of these 24 beams (green circles) as well as the remaining 36 beams (red circles) after the rotation of the Omega facility. Figure 1b also shows the highly symmetrical 24-beam direct-drive configuration (black circles) provided by Skupsky and Lee^31^. This is the original 24-beam Omega facility where 12 laser beams per hemisphere are located at the rings with polar angles θ_1_ (4 beams) and θ_2_ (8 beams). As can be seen, the green and black circles differ only by small angles, with the largest differences smaller than 5^°^. Thus, 24 beams of the 60-beam configuration can be selected and represent a configuration similar to the original Omega facility while the remaining 36 laser beams form a second independent configuration. It will be shown that these two subjacent configurations can each provide quite small illumination non-uniformity below 1%, allowing them to be used separately. It has been shown, a long time ago by Skupsky and Lee^31^, that the root-mean-square (rms) illumination non-uniformity σ can be expressed as the sum of the squares of the non-uniformities associated with each Legendre polynomial mode P_l_. For a set of N beams located at the angular directions Ω_i=1, N_, the expression for σ reads as:

1 $$\sigma^{2} = \mathop \sum \limits_{l \ne 0}^{{}} \sigma_{l}^{2} ;\;with\;\sigma_{l}^{2} = \left| {E_{l} /E_{0} } \right|^{2} \left[ {\left( {2l + 1} \right)\mathop \sum \limits_{i} \mathop \sum \limits_{j} P_{l} \left( {\Omega_{{\varvec{i}}} \cdot \Omega_{{\varvec{j}}} } \right)\frac{{W_{i} W_{j} }}{{\left( {\sum W_{k} } \right)^{2} }}} \right];$$

and, for N beams with the same energy and deposition patterns (w_i=1:N_ = w_0_), σ_*l*_ =|E_*l*_/E_0_| G_*l*_, where G_*l*_ is the geometrical factor

2 $$G_{l}^{{}} = \left[ {\left( {2l + 1} \right)\mathop \sum \limits_{i} \mathop \sum \limits_{j} P_{l} \left( {\Omega_{{\varvec{i}}} \cdot \Omega_{{\varvec{j}}} } \right)} \right]^{1/2} /N^{{}}$$

Equation (1) shows that the non-uniformity associated with each Legendre mode, σ_l_, can be factorized as the product of two terms: the single beam factor ( |E_l_/E_0_| ) that takes into account the laser intensity profile as well as the details of the laser energy deposition, and the geometrical factor ( G_l_ ) that depends only on the number of beams (N) and their geometrical distribution (Ω_i_). Moreover, in the case that each beam, characterized by an angular position Ω_i_, has an associated opposite beam at the position Ω_j_ = -Ω_i_—as in our case—the geometrical factors of all odd modes vanish^31^. Table 1 reports the geometrical factors G_l_ for the first 10 even Legendre modes. The lower dominant modes, whose *l* increases with beam number N approximately as π N ^1/2^/2 as expected^31^, are highlighted in bold in the table. The first column gives the geometrical factors calculated for the 24-beam configuration provided by Skupsky and Lee that corresponds to the original 24-beam Omega. Their values are quite similar to those of the 24-beam subset of Omega-60. The 36-beam configuration provides slightly smaller geometrical factors in comparison with the 24-beam configuration, and the full 60-beam Omega facility provides the best results, with all Legendre modes lower than *l* = 10 vanishing.

**Table 1:** First even geometrical factors G_l_ for the configurations of 24 beams of Skupsky and Lee, the 24- and 36-beam subsets, and the full 60 Omega beams.

l	24 S&L	24 beams	36 beams	60 beams
2	0.0	0.0	0.0	0.0
4	0.07	0.21	0.14	0.0
6	0.30	0.43	0.28	0.0
8	**1.71**	**1.59**	1.06	0.0
10	1.50	1.58	**1.40**	**1.25**
12	1.56	1.59	1.07	0.20
14	0.0	0.59	0.39	0.0
16	1.18	1.09	1.00	0.92
18	3.05	2.84	2.56	2.32
20	0.44	1.08	0.90	0.72

Significant values corresponding to the lower dominant modes are in bold.

**Figure 2:**
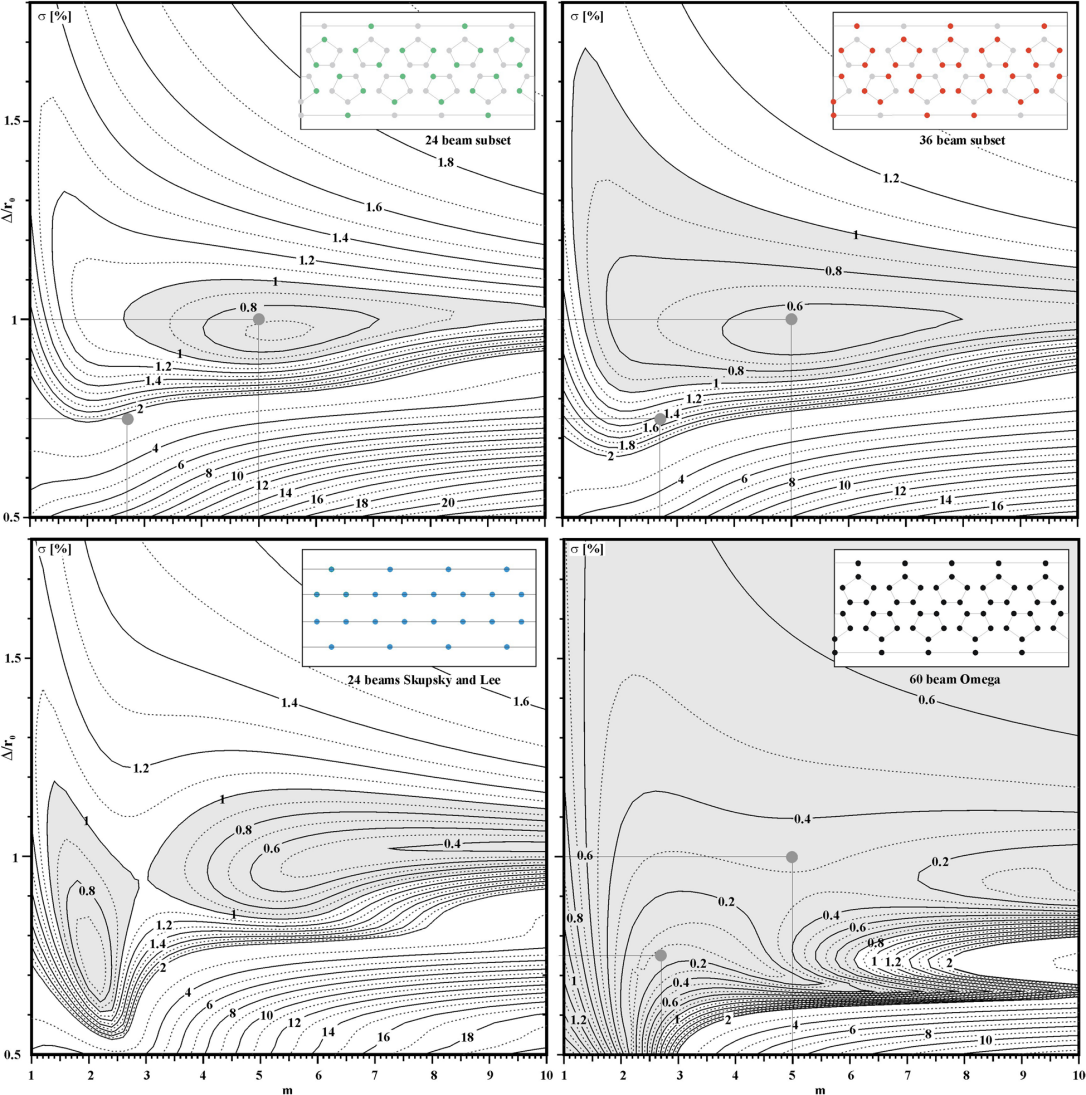
Rms illumination non-uniformity (σ [%]) for the 24, 36 and 60 laser-beam configurations of the Omega facility as a function of the laser focal spot parameters Δ/r_0_ and m. The shaded areas indicate where σ < 1%.

A parametric study has been performed to calculate σ for the four configurations: the 24-beam configuration of Skupsky and Lee^31^, the 60 beams of Omega, and the 24- and 36-beam subsets of Omega. Figure 2 plots the rms illumination non-uniformity ( σ ) averaged over the surface of a solid sphere of radius r_0_, numerically calculated assuming that the N laser beams are directed toward the centre of the sphere^32–36^. Each laser beam is characterized by its direction of irradiation (Ω_i_) and by a super-Gaussian intensity profile I(r) = I_0_ exp[-(r/Δ)^m^], where Δ is the half width at 1/e radius and m the exponential factor. The illumination uniformity provided by a given irradiation scheme is given in terms of the root mean square (rms) deviation σ of the total laser intensity generated by the N laser beams:

3 $$\sigma = \left[ {\frac{1}{{4\pi <I>^{2} }}\mathop \smallint \limits_{0}^{2\pi } \mathop \smallint \limits_{0}^{\pi } \left( {\mathop \sum \limits_{i = 1}^{N} I_{i} \left( {\theta ,\varphi } \right) - <I>} \right)^{2} {\text{sin}}\left( {\uptheta } \right)d\theta d\varphi } \right]^{1/2}$$

where < I > is the average intensity and the integral covers the whole sphere. Evidently, the illumination model does not take into account beam refraction, laser parametric instabilities or cross-beam energy transfer (CBET) between different beams but represents only the intrinsic illumination uniformity.

Figure 2 plots σ as a function of the relative laser spot size Δ/r_0_ and the super-Gaussian exponential factor m. The parametric space varies between 0.5 < Δ/r_0_ < 1.8 and 1 < m < 10. As can be seen, the illumination non-uniformity is smaller than 1% for all configurations within the relatively large parameter space indicated by the shaded areas. This confirms that the two subsets of 24 and 36 laser beams can be separately considered as direct-drive "facilities". Moreover, all these configurations exhibit a small rms non-uniformity for laser intensities characterized by an exponential factor of around m = 5 and for Δ/r_0_ ≈ 1. Thus the minimum illumination non-uniformity provided by the 60-beam configuration (≈ 0.1%) is better than the 36 laser-beam configuration (≈ 0.6%), which is slighter better than the 24-beam configuration (≈ 0.7%). It may be noted that the illumination non-uniformity for the 24-beam configuration of Skupsky and Lee^31^ provides a smaller minimum of about 0.5% and maintains the non-uniformity σ below 1% over a broader region in parameter space.

For direct drive, the drive uniformity depends on the uniformity of deposited laser energy, which depends on the convolution of the inverse bremsstrahlung absorption with the time-varying target parameters. In particular, the density profile changes as the plasma expands outwards and as the critical radius (defined as the radial position of the critical density n_c_ ~ 10^21^/λ^2^ [µm]) decreases due to the implosion. While not included in the illumination model described above, these effects are included in the 2D hydrodynamics code SAGE^37,38^, which also takes 3D refraction into account, depositing laser energy continuously along the ray trajectories. To compare the illumination model and the SAGE model, we consider a benchmark calculation that simulates a 4-mm-diameter CH target implosion experiment performed on the NIF^39^ and known as "Orange". In this experiment, the laser pulse presents a ramp and a constant plateau at 390 TW power during 3.7 ns. In the calculations presented here, the implosion is driven by the different direct drive geometries, i.e. either 24, 36 or 60 beams. In all three calculations, the same total energy is considered and the only difference is in the configuration of the beams, all other things being equal.

The illumination model results for rms non-uniformity are compared with the SAGE results for all three geometries in Table 2, for two different super-Gaussian laser focal spots (Δ/r_0_ = 0.75, m = 2.7) and (Δ/r_0_ = 1, m = 5). The time-integrated laser absorption calculated by SAGE and the laser-target coupling calculated by the illumination model are also given; for the illumination model the laser-target coupling efficiency is defined as the ratio of the laser intensity integrated on the target surface to the incident intensity integrated over all the N beam focal spots.

**Table 2:** Comparison of uniformity predictions from the illumination model and SAGE for the 24, 36, and 60 beam configurations for two different super-Gaussian focal spots.

focal spot	model	laser absorption [%]	laser-target coupling [%]	24 beams σ rms [%]	36 beams σ rms [%]	60 beams σ rms [%]
Δ/r_0_ = 0.75, m = 2.7	Illumination		93.1	2.15	1.44	0.12
	SAGE	90.9		1.93	1.30	0.25
Δ/r_0_ = 1, m = 5.0	Illumination		88.1	0.71	0.53	0.35
	SAGE	84.5		0.51	0.35	0.13

The time-integrated absorption (for SAGE) and laser-target coupling efficiency (illumination model) are also given.

It is seen that the SAGE calculations are consistent with the illumination model on the two configurations underlying the nominal 60-beam configuration, showing similar trends and confirming the compatibility of the 24- and 36-beam configurations with uniform direct drive.

As stated above, the proposed configuration cannot be tested on Omega because the desired 24- and 36-beam subsets cannot be given independent pulse shapes. Omega does have provision for independent pulse shapes in 20- and 40-beam subsets, implemented at the first splitting stage in the laser system where one of the three 20-beam “legs” can be fed by a different driver line. However, the beam locations on target are highly irregular without any geometric symmetry. Similar SAGE calculations were reported for this in Ref. 48, where the beams were not pointed to target centre as in the present work but were repointed to improve the uniformity. With optimum repointing, the 40-beam set gave a reasonable uniformity of 1.2%, but the 20-beam set gave an excessive 3.9%.

### Laser-capsule scaled to the Omega facility

Now that the uniformity of the 24- and 36-beam subsets has been established, we investigate designs that take advantage of two-step zooming. The most likely implementation would be to use the 24-beam configuration for the foot pulse, using a large laser spot to assemble the thermonuclear fuel, and the 36-beam configuration with a smaller laser spot to send a relatively short, high-powered, high-intensity pulse to assist the ignition of the fuel. (Alternatively, one could use 36 beams followed by 24; this would produce better early-time uniformity but a lower intensity on the converging fuel around the time of ignition.) We have chosen to start with a high-gain thermonuclear target designed at the megajoule scale, and then scale this down to be consistent with the energy and power levels of the 24-beam configuration of Omega (maximum laser energy of 0.5 kJ and maximum power of 0.5 TW per beam). A second 36-beam pulse is then added subject to the same constraint. Results for two-step zooming at the Omega energy level should provide insight into the required energy at which an intermediate-scale system would produce ignition.

**Figure 3:**
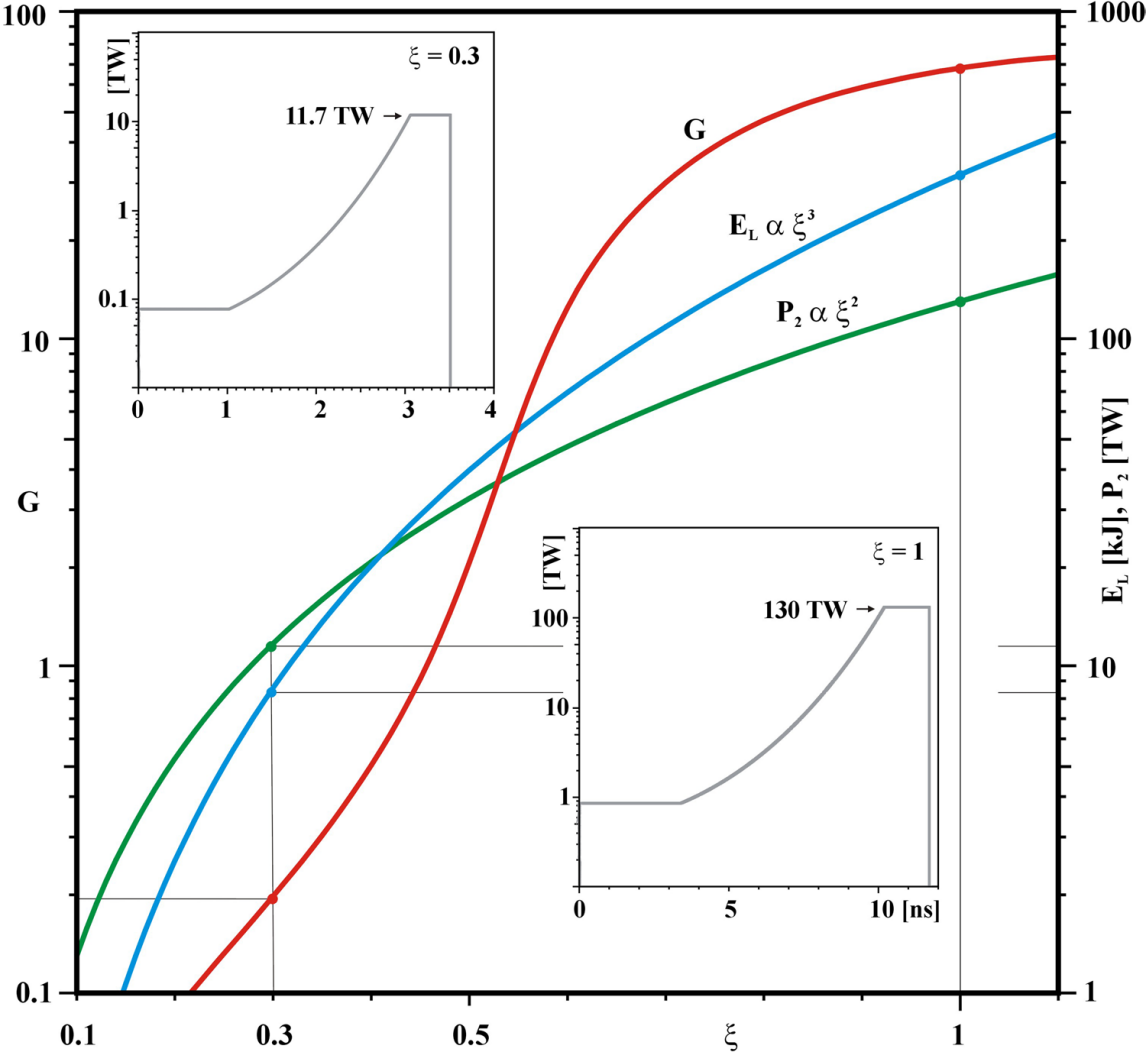
Absorbed laser energy (E_L_), peak laser power (P_2_) and energy gain (G) as a function of the homothetic factor ξ. In the insets are shown the two temporal absorbed laser profiles P(t) for the cases characterized by ξ = 1 and ξ = 0.3.

The megajoule-scale target is a spherical layer of plastic ablator (CH, ρ_CH_ = 1.07 g/cm^3^) of thickness 16.6 μm with an outer radius of 976.5 μm that contain the cryogenic deuterium–tritium (DT) fuel, a layer of thickness Δ_DT_ = 120 μm which corresponds to a DT mass of m_DT_ = 300 μg. The central part, whose radius is r_0_ = 840 μm, contains a residual DT gas at a density of 0.3 g/cm^3^. The capsule is thus characterized by an initial aspect radio A = r_0_ / Δ_DT_ = 7. The laser pulse is designed to have a pre-pulse (the foot) of power P_1_ = 0.85 TW kept constant for a time t_1_, as shown in the bottom inset of Fig. 3. Then the absorbed power increases to the power P_2_, reached at time t_2_, following a Kidder-like^40^ power law P(t) = P_0_ [1-(t/20 ns)^2^]^β^. This maximum power P_2_ is maintained constant for a time of 1.5 ns and this last part is called the drive. The two parameters P_0_ and β are determined by the two conditions P(t_1_) = P_1_ and P(t_2_) = P_2_.

Hydrodynamics calculations have been performed using the 1D numerical code Multi-IFE^41^. The code assumes electron and ion plasma temperatures, Spitzer heat conduction^42^ harmonically limited to 8% of the free streaming limit^43^, multi-group radiation transport, and tabulated equations of state. Usually, a 3D ray-tracing package is used to propagate the laser light (3ω, λ = 0.35 μm) while the absorption is modeled by inverse-bremsstrahlung. Nevertheless, in order to be independent of the laser focal spot, in this first set of calculations a 1D ray-tracing model is assumed where laser light propagates along a radius until the critical density, reflects and returns along its path. Furthermore, in this first set of calculations, at each time-step the code adjusts the incident laser power to assure that the absorbed laser power coincides with the input laser pulse, thus P_abs_(t) = P(t). Several thousands of 1D calculations have been performed by randomly varying the three pulse parameters t_1_, t_2_ and P_2_. From these calculations a laser pulse was selected, characterized by t_1_ = 3.4 ns, t_2_ = 10.2 ns, and P_2_ = 130 TW. This pulse was chosen because it provides a robust configuration far above the ignition threshold. This laser-capsule design delivers a thermonuclear energy of 21.3 MJ for an absorbed energy of 317 kJ, corresponding to an energy gain G (relative to the absorbed laser energy) of 67 and a maximum implosion velocity of 357 μm/ns.

In order to match the energy and power delivered by Omega, this target design is scaled down by using the homothetic transformation^44,45^ where dimensions and times scale proportionally to the factor ξ, laser power scales as ξ^2^, and consequently energy (P t) and mass (P t /c^2^) scale as ξ^3^ while velocity α (r/t) and density α ( m/r^3^) are conserved. Figure 3 shows the energy gain (G), the laser energy (E_L_), and the maximum laser power of the drive (P_2_) as a function of ξ.

**Figure 4:**
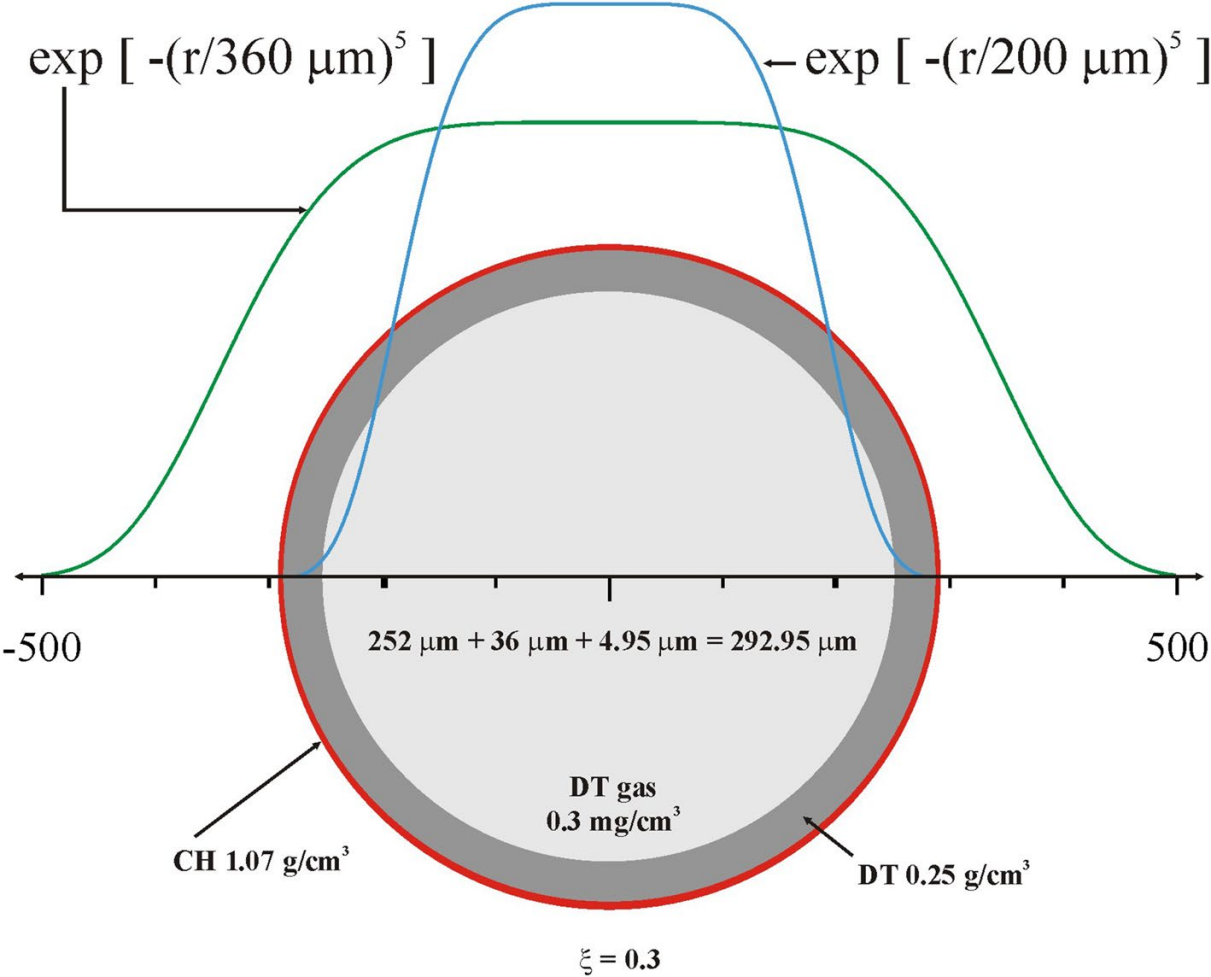
Laser and target characteristics, scaled down from the megajoule design using the homothetic factor ξ = 0.3, to match the energy and power limits of the 24-beam Omega subset. Two laser intensity profiles characterized by Δ = 200 μm and Δ = 360 μm with the exponential factor m = 5 are also shown.

Since laser power scales as ξ^2^, the maximum laser power associated with the homothetic factor ξ = 0.3 is 11.7 TW, which almost matches the maximum power available on Omega for the 24-beam configuration. Thus, ξ = 0.3 is chosen to scale the megajoule target design to one characterized by an outer radius of 292 μm, hereafter the nominal target design. The laser pulse is characterized by a low-power foot with P_1_ = 0.08 TW that is steady for the first ns (t_1_ = 1 ns). The power then increases until it reaches the power P_2_ = 11.7 TW at time t_2_ = 3.06 ns after which it remains constant for 0.45 ns. Hereafter, a 3D ray-tracing package has been used to propagate the laser light through the plasma. The focal spot is assumed to be super-Gaussian with Δ = 360 μm and exponent m = 5^46,47^ and the absorbed laser energy is reduced to 8.6 kJ. The capsule and laser focal spot dimensions are shown in Fig. 4.

### Ignition assisted by a secondary laser pulse

**Figure 5:**
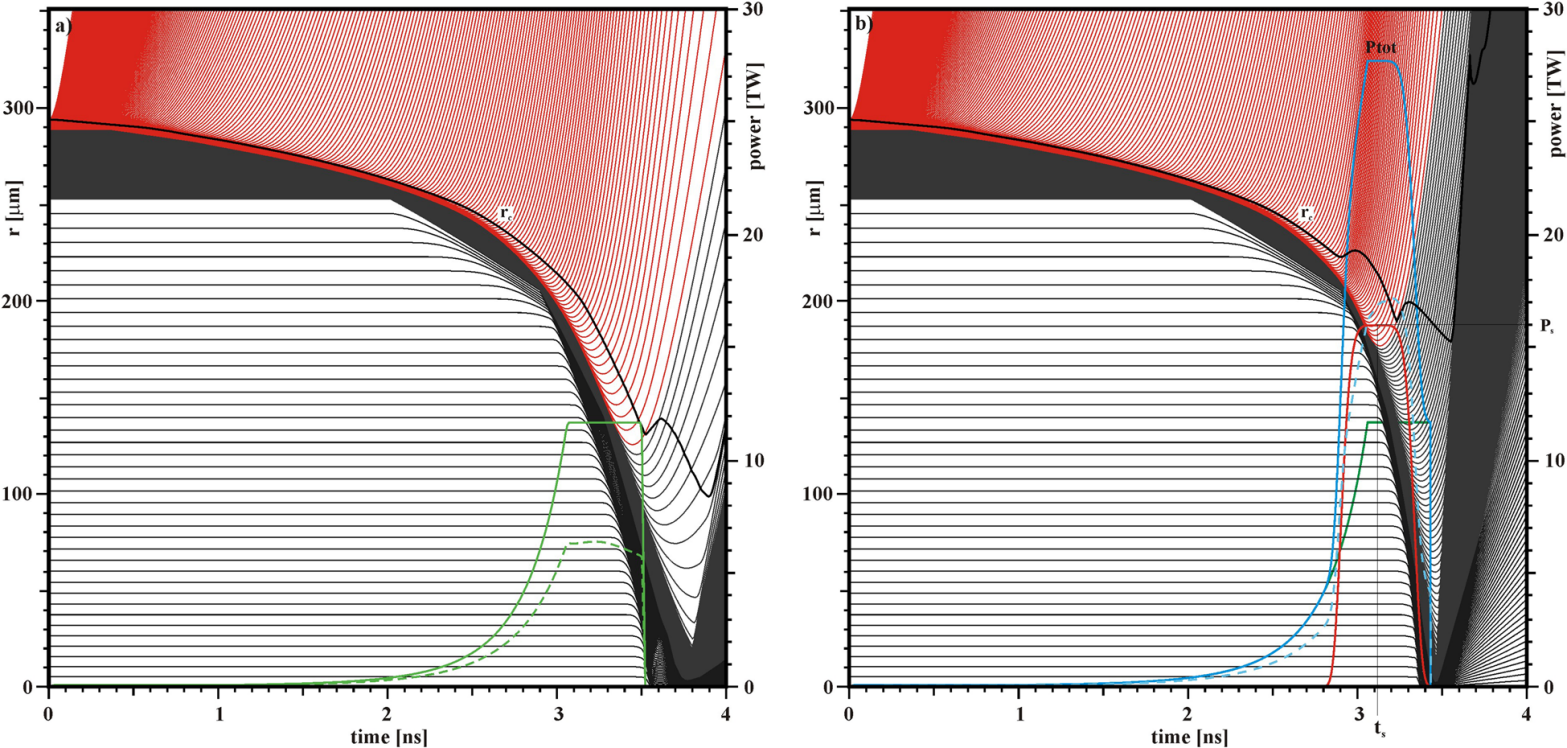
(**a**) Hydrodynamic calculation of Lagrangian trajectories for the reference case. (**b**) The same but for a case with ignition assisted by a secondary laser pulse. The green curve gives the primary laser power, the red curve the secondary laser power, the blue curve the total incident laser power, and the dashed curves the total absorbed laser power. The black line (r_c_) shows the position of the critical density.

A hydrodynamic simulation of the nominal target associated with the homothetic factor ξ = 0.3 provides the evolution of the Lagrangian cells as a function of time, shown in Fig. 5a together with the incident and absorbed laser power. In this case, the absorbed laser energy is 8.6 kJ and the fusion energy produced is 0.08 kJ, which corresponds to a 1D energy gain G = 0.01. Figure 5a also shows the time evolution of the radius r_c_ (black line) where the density is equal to the critical density ρ_c_ = 1.865 10^−3^ (A/Z)/ λ^2^, where ρ_c_ is in g/cm^3^ and λ in μm.

A second laser pulse is used to assist the ignition and the thermonuclear burn propagation in order to generate high energy gain. This second laser pulse has an almost square temporal profile: P(t) = P_s_ exp{− [(t–t_s_)/225 ps]^6^} characterized by the two parameters P_s_ and t_s_. The maximum laser power is P_s_ and the pulse is centered at the time t_s_ with a full 1/e width of 450 ps, which approximately matches the duration of the main drive of the first laser pulse. As shown in Fig. 5(a) the critical radius r_c_ is reduced from almost 240 μm to 150 μm during the main drive. Thus, a relatively small laser focal spot characterized by a 1/e radius of 200 μm and a super-Gaussian exponent m = 5, as shown in Fig. 4, is associated with the secondary laser pulse. Figure 5b shows the implosion trajectories for the case with a second laser pulse centred at t_s_ = 3.12 ns and with a maximum laser power P_s_ = 16 TW. The total incident laser power provided by the sum of the two pulses reaches about 28 TW, the incident laser energy is 14.3 kJ, and the absorbed laser energy is 8.6 kJ. As can be seen, in this case the critical radius varies approximately between 240 μm and 190 μm during the main drive. This configuration generates an output fusion energy of 18.3 kJ, which corresponds to a 1D energy gain G of 2.1 relative to absorbed energy (1.3 relative to incident energy). The use of a secondary laser pulse with higher power and a smaller focal spot as shown in Fig. 5b is similar to what is conceived for shock ignition^29^. An Omega-like system supporting the 24/36 beam sets would be able to explore shock ignition under conditions of good uniformity.

**Figure 6:**
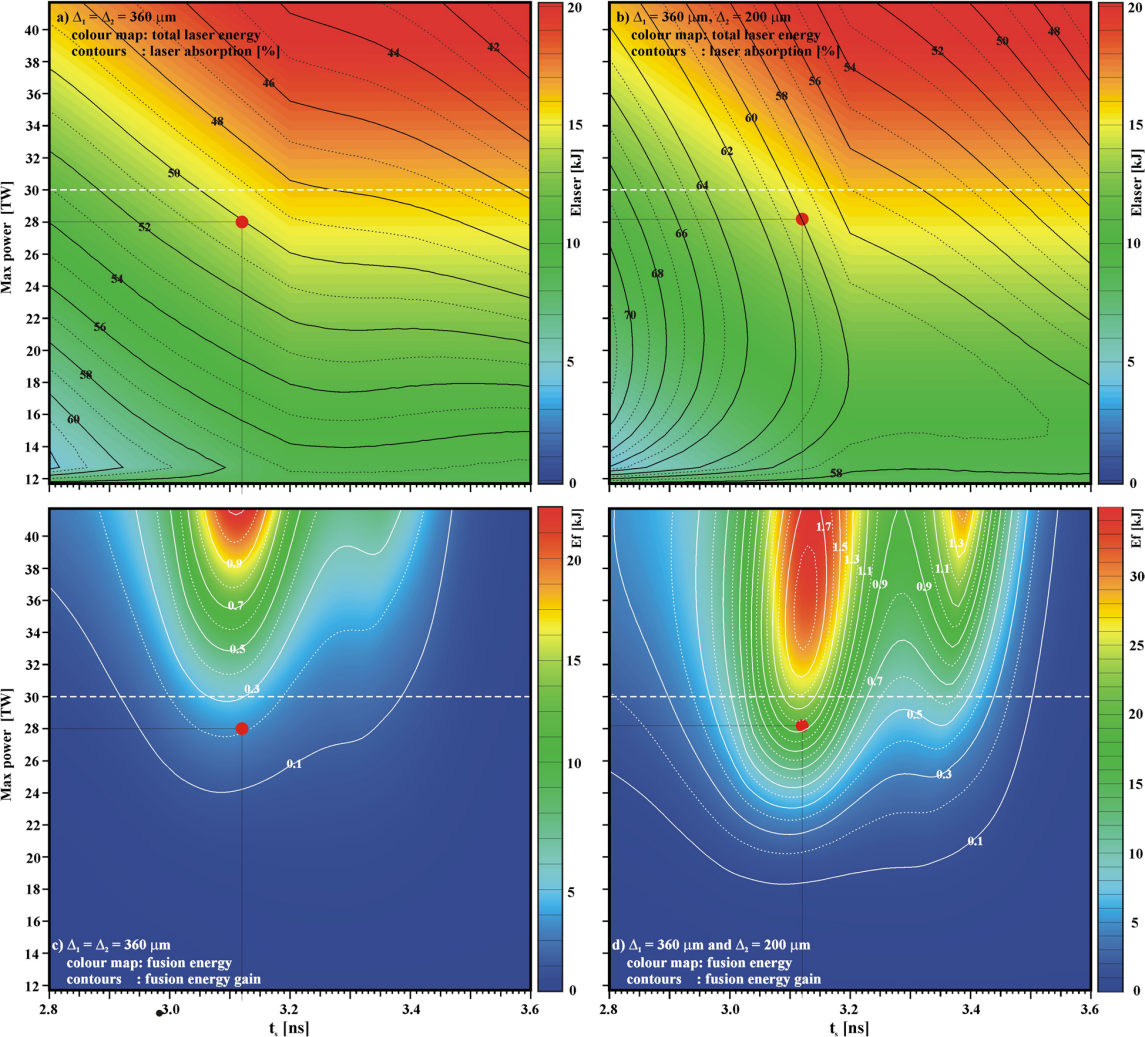
(**a**) total laser energy (colour maps) and laser absorption (contour lines), (**c**) output fusion energy (colour maps) and the energy gain relative to the incident laser energy (contour lines) as functions of the timing t_s_ of the second pulse and the maximum total power for the calculations using the larger focal spot (Δ_1_ = Δ_2_ = 360 μm). The frames (**b**) and (**d**) refer to the cases using the smaller focal spot (Δ_2_ = 200 μm) for the second pulse. The dashed horizontal lines indicate the maximum power (30 TW) available on Omega.

The performance of this configuration, which assists the ignition with an additional laser pulse, also depends on the time t_s_ and the power P_s_ of this additional pulse. A parametric study has been performed in order to assess the sensitivity of the system when varying the two parameters t_s_ and P_s_ between 2.8 ns and 3.6 ns and 0 to 30 TW, respectively. Thus the maximum power—superposition of the two pulses—varies from 11.7 TW, corresponding to P_s_ = 0, to 41.7 TW for P_s_ = 30 TW. Moreover, two sets of calculations were performed: a first set that uses the same—relatively large—super-Gaussian laser focal spot with Δ = 360 μm and an exponential factor m = 5 for both laser pulses, while in a second set a smaller focal spot (Δ = 200 μm, m = 5) is associated with the second, powerful laser pulse. Figure 6a and b show the total incident laser energy (colour maps) as a function of the two parameters t_s_ and the maximum power. In Fig. 6a the large focal spot was used for both laser pulses, while in Fig. 6b the smaller laser focal spot was used for the secondary laser pulse. In the same figures, the total laser absorption [%] is also shown by contour lines. As can be seen, the total laser absorption is significantly increased when the smaller focal spot is used (b) compared to the opposite case (a). As an example, for the case previously mentioned corresponding to the parameters P_s_ = 16 TW and t_s_ = 3.12 ns (red circle), the laser absorption increases from 50% with the larger laser focal spot to 60% when the smaller spot is used. Both images 6(c) and 6(d) show the output fusion energy (colour maps) and the energy gain (contour lines). It is seen that the cases with the larger focal spot 6(c) never provide an energy gain larger than one. In contrast, when the laser absorption is increased using the smaller focal spot 6(d), energy gains larger than one are generated in a fairly large parameter space.

It is worth noting that in all these calculations the total laser energy involved never exceeds the 30 kJ theoretically available at the scale of the Omega facility. However, in a fairly large part of parameter space the production of a gain greater than one requires a total laser power greater than the 30 TW maximum of the Omega facility. Thus, in these calculations, it is the laser power that limits the production of energy gain rather than the total laser energy. 1D calculations usually overestimate the thermonuclear energy in ICF. But these results point out the possibility of approaching and studying the self-ignition threshold with mid-scale Omega-like facilities delivering less than 50 kJ.

## Conclusions

In this work it has been shown that the 60-beam Omega configuration can be divided into two separate sets of 24 and 36 laser beams, respectively, each allowing for uniform direct-drive implosions. A simple illumination model and hydrodynamics simulations using the code SAGE with 3D ray tracing both obtained consistent results with the non-uniformity below 1%. This opens the possibility of two-step zooming on a future 60-beam laser system in which the two sets deliver different laser temporal pulse shapes and produce different focal-spot profiles on target. In the scenario considered here, the 24-beam set produces a relatively large laser spot, comparable to the initial target size, and delivers a standard shaped pulse that increases from a low-intensity foot to high intensity at the implosion time. The 36-beam set is focused to a smaller spot, for greater absorption efficiency, and timed when the imploding target is approaching peak compression to provide a more powerful main drive.

To investigate this concept, a high-gain megajoule-scale direct-drive target was scaled down to a level consistent with Omega limits on power (0.5 TW / beam) and energy (0.5 kJ / beam). The laser parameters and pulse shape were applied to the 24-beam set. The 36-beam set was used to deliver higher powers in shorter pulses timed to match the main-drive portion of the 24-beam pulse shape, and was focused to a smaller spot size (1/e radius 200 µm rather than 360 µm). A parametric study was carried out in which the peak power and timing of the 36-beam set were varied to identify the conditions in which a gain greater than one would be obtained. Two series of 1D simulations were included with different spot sizes for the 36-beam set (200 µm and 360 µm). It was found that gains greater than one were possible for the smaller 36-beam spot size and laser power levels somewhat higher than are available on Omega.

These results are regarded as optimistic because they were based on ideal 1D simulations. A multi-dimensional analysis is required that takes into account (at least) the low-mode asymmetries generated by each configuration. However, the results demonstrate the concept of using a 60-beam laser system with the 24- and 36-beam subsets providing a two-step zooming. A future intermediate-energy laser system, with energy greater than Omega but less than a megajoule, could take advantage of this concept.

## Data Availability

The data that support the plots and findings of this paper are available from the corresponding author upon reasonable request.
